# Current Practice and trends in echocardiography in Critical Care, Anesthesiology and Emergency Medicine

**DOI:** 10.2174/157340311798220476

**Published:** 2011-08

**Authors:** Paulo Marcelino

Echocardiography is a valuable diagnostic tool allowing visualization of heart chambers for a complete anatomical and functional evaluation. In the past years there was a clear trend toward its use in other than cardiology areas, like intensive care and anesthesiology. In the Intensive Care its use is now widely accepted, as the concepts on hemodynamic monitoring evolved. This evolution is related to the criticism on pulmonary artery catheter use and its relation with both morbidity and mortality. The use of invasive diagnostic tools is therefore to be avoided but alternatives should also be available. Echocardiography equipment´s evolution toward better downsizing and lower price made the technique even more assessible in the past decade. The use of echocardiography offers the intensive care phisician a tool for a quick and comprehensive diagnosis of the critically ill; also, the examination is fast, does not require the involvement of other health personnel or disposable materials. The information is assessed in real time and obtained based on direct visualization of the heart. Specific issues for the intensive care are also studied using echocardiography. Among them is the sepsis related cardiomyopathy. In anesthesiology the use of echocardiography is quite established, especially in cardiac surgery monitoring. However echocardiography is also a valuable tool in non-cardiac surgery. The evaluation of volume status, left ventricular performance or right heart chambers monitorng are all significant information, not provided by other monitoring tools. The expanded use of echocardiography in non-cardiac surgery should be the next step in this evolution, and many anesthesiologists are using it. Specific monitoring protocols for specific situations must be implemented. The assessment of the patients on the emergency room using echocardiography is an area of growing interest. Contrast echocardiography is a good example. Although patients with suspected heart disease are more often evaluated, the possibilities of echocardiography go far beyond this type of patients. Many features of the approach used in the intensive care can be applied, as well as other potential uses beyond strict heart imaging. Echocardiography is now an image technique used by several non cardiologists specialists. A specific knowledge in this different settings is expected, emerging from its use in distinct disciplines.

